# Evaluation of patients with a recent clinical fracture and osteoporosis, a multidisciplinary approach

**DOI:** 10.1186/1471-2474-9-109

**Published:** 2008-08-05

**Authors:** Bianca Dumitrescu, Svenjhalmar van Helden, Rene ten Broeke, Arie Nieuwenhuijzen-Kruseman, Caroline Wyers, Gabriela Udrea, Sjef van der Linden, Piet Geusens

**Affiliations:** 1 Department of Internal Medicine, Subdivision of Rheumatology, University Hospital, Maastricht, The Netherlands; 2 Department of Rheumatology, University of Medicine and Pharmacy 'Carol Davila' Bucharest, Romania; 3 Department of Trauma Surgery, University Hospital, Maastricht, The Netherlands; 4 Department of Orthopaedic Surgery, University Hospital, Maastricht, The Netherlands; 5 Department of Internal Medicine, Division of Endocrinology, University Hospital, Maastricht, The Netherlands; 6 Epidemiology, Maastricht University, The Netherlands; 7 Biomedical Research Centre, University Hasselt, Belgium

## Abstract

The aetiology of osteoporotic fractures is multifactorial, but little is known about the way to evaluate patients with a recent clinical fracture for the presence of secondary osteoporosis.

The purpose of this study was to determine the prevalence of contributors to secondary osteoporosis in patients presenting with a clinical vertebral or non-vertebral fracture. Identifying and correcting these contributors will enhance treatment effect aimed at reducing the risk of subsequent fractures.

In a multidisciplinary approach, including evaluation of bone and fall-related risk factors, 100 consecutive women (n = 73) and men (n = 27) older than 50 years presenting with a clinical vertebral or non-vertebral fracture and having osteoporosis (T-score ≤-2.5) were further evaluated clinically and by laboratory testing for the presence of contributors to secondary osteoporosis.

In 27 patients, 34 contributors were previously known, in 50 patients 52 new contributors were diagnosed (mainly vitamin D deficiency in 42) and 14 needed further exploration because of laboratory abnormalities (mainly abnormal thyroid stimulating hormone in 9). The 57 patients with contributors were older (71 vs. 64 yrs, p < 0.01), had more vertebral deformities (67% vs. 44%, p < 0.05) and a higher calculated absolute 10-year risk for major (16.5 vs. 9.9%, p < 0.01) and for hip fracture (6.9 vs. 2.4%, p < 0.01) than patients without contributors. The presence of contributors was similar between women and men and between patients with fractures associated with a low or high-energy trauma.

We conclude that more than one in two patients presenting with a clinical vertebral or non-vertebral fracture and BMD-osteoporosis have secondary contributors to osteoporosis, most of which were correctable. Identifying and correcting these associated disorders will enhance treatment effect aimed at reducing the risk of subsequent fractures in patients older than 50 years.

## Background

Clinical vertebral and non-vertebral fractures are the most frequent fractures in patients presenting to the emergency ward of the hospital with a fracture [1]. After such fracture, patients are at increased risk for subsequent fracture and guidelines on osteoporosis advocate to evaluate patients presenting with a fracture in order to consider treament to reduce the risk of subsequent fractures [2].

One aspect of care identified within the management of fracture patients is the existence of contributors to secondary causes of bone loss [3]. Effective therapy requires that these contributors be recognised and when present managed appropriately [3,4]. If these conditions, however, are not recognized, treatment may be suboptimal or ineffective [5,6].

Apart from bone mineral density (BMD)-osteoporosis (T-score less than or equal to -2.5) [4,7,8] many risk factors are related to fracture risk, independently of BMD, such as clinical risk factors [9], fall risks [10], prevalent morphometric vertebral fractures (MVF) [11] and secondary osteoporosis [12]. There is increasing evidence that secondary osteoporosis is more prevalent than initially thought, not only in males, but also in females [13], but the true prevalence of contributors to secondary osteoporosis is unknown and no consensus regarding its evaluation is available [14].

Published data from referral centres for evaluation of osteoporosis indicate that 32 to 37% of women with low BMD have a history of other diseases or medications known to contribute to osteoporosis [3,15]. From 20% up to 64% of patients had previously unknown secondary causes of osteoporosis that were only identified by laboratory testing [5]. In a study of patients with a clinical fracture, a high prevalence of contributors to secondary osteoporosis (77%) was reported, but the study included only a limited number of patients with measured low BMD [16]. In a study of patients with a hip fracture, 80% had secondary causes of bone loss, mainly related to disturbed calcium and vitamin D homeostasis [6]. To date, we lack studies on the prevalence of contributors to secondary osteoporosis in other fracture populations.

The purpose of this study was to determine the prevalence of contributors to secondary osteoporosis, in the context of other bone- and fall-related fracture risks in patients presenting with a clinical vertebral or non-vertebral fracture and with a low BMD. Identifying and correcting contributors will enhance treatment effect aimed at reducing the risk of subsequent fractures.

## Methods

**Figure 1:**
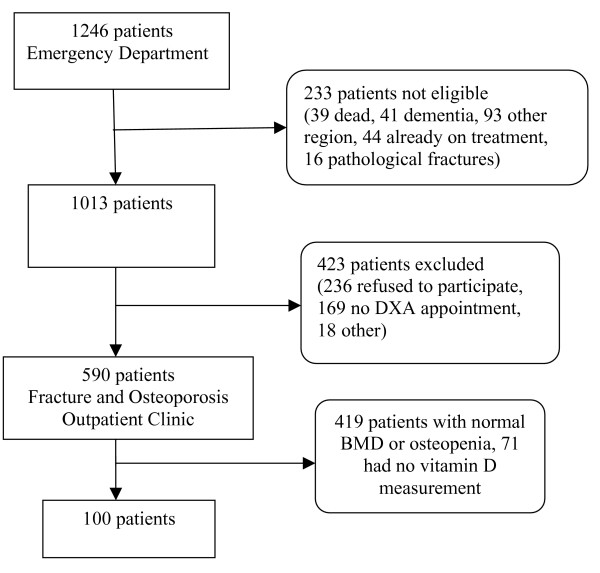
Flow chart of patients included in the study (see text for details) in one year.

In this prospective observational study, 100 consecutive and consenting patients older than 50 years, who presented between April 2005 and April 2006 with a clinical fracture at Maastricht University Hospital in the Netherlands, were included. After receiving medical treatment for the fracture, patients had a consultation with the fracture nurse. The fracture nurse provided information about the study and invited the patients to the Fracture and Osteoporosis Outpatient Clinic. Patients already on osteoporosis treatment (44/1246, 4% of all) or with pathological fractures due to malignancy or Paget's disease of bone were excluded from the analysis. Patients who agreed to participate were further referred to the program. Patients with osteoporosis according to World Health Organization (WHO) criteria for BMD [4] and in whom all laboratory data were available were included in the present study (Figure 1). This group was part of the evaluation of all consecutive patients presenting with a clinical fracture, of whom 35% had osteoporosis and 44% had osteopenia [1]. The medical ethical committee of the University Hospital Maastricht approved the study (MEC 03-194-5).

BMD in the left or right hip and the lumbar spine was determined using dual X-ray absorptiometry (DXA) with Hologic QDR 4500 Elite. Diagnosis of osteoporosis was based on the WHO criteria for BMD [4], as provided by the manufacturer for women and men. Patients were classified according to the lowest value of T-score in either total hip or spine.

All patients were interviewed for bone-related risk factors for fracture (previous non-vertebral and vertebral fractures, mother with fracture, body weight <60 kg, severe immobility, use of glucocorticoids) and fall-risk factors (falls in the past 12 months, use of assistive devices, sedative medication, activities of daily living, mobility, impaired vision, articular complaints, urine incontinency), according to the Dutch guidelines on osteoporosis [2] and fall prevention [10]. Additionally, data about vitamin D status (regular sun exposure, dietary intake and supplements), calcium intake [17,18], height, history of height loss [19] and a description of the circumstances leading to the fracture (with specification of fall from a standing height or other trauma) were recorded.

Patients with T-scores ≤-2.5 were given a pre-planned set of laboratory tests that included erythrocyte sedimentation rate (ESR), haemoglobin, leucocytes and serum levels of creatinine, calcium, albumin, alkaline phosphatase, 25-OHD_3 _and TSH. Calcium and creatinine were measured in a 24-hour urine collection. All laboratory analyses were performed in the same laboratory. Patients with osteoporosis and having the full set of evaluation were sent for a consultation with either a rheumatologist or an endocrinologist. The specialist decided further investigation and treatment. When clinically appropriate, additional tests were performed. The diagnosis of contributors to secondary osteoporosis was based on all data from the medical files. Renal insufficiency was defined with the cut-off value of creatinine clearance ≤40 using the Cockroft Gault formula [20]. Vitamin D status was defined as severely deficient when values were ≤30 nmol/L, deficient when between 30 and ≤50 nmol/L, insufficient when values were between 50 and ≤75 nmol/L [21,22] and abnormally high when above 220 nmol/L [23]. Exploration for hypogonadism in men was considered when a morning serum testosterone level was below 12 nmol/L [24] and for thyroid disorders when TSH were outside the reference ranges (0.4–3.5 mU/L). Hyperparathyroidism was diagnosed when serum parathyroid hormone (PTH) levels were above 5.5 pmol/l. Further exploration for hypercalciuria was considered when the total urinary calcium in a 24 hours collection exceeded 7 mmol/d and creatinuria indicated an appropriate collection (between 4.5 – 13.3 mmol/hour) [25]. According to clinical judgement, patients suspected to have lactose intolerance had a lactose tolerance test [26].

Vertebral fracture assessment (VFA) [27,28] by single X-ray absorptiometry on the lateral spine images was performed to identify the presence of morphometric vertebral deformities (MVD). Images were saved in a digital format. Physician Viewer software (Hologic, USA) provided the tools necessary to perform quantitative vertebral morphometry. Visual assessment and measurements of the anterior, posterior and mid heights from T4 to L4 were performed twice, by a trained rheumatologist (BD). These assessments were inputted into a database. The observer was blinded from the results of the first measurements. The intra-observer coefficient of variation (ICC) at vertebral level for heights was 0.917 (95% confidence interval (CI): 0.905–0.930, Cronbach's alpha 0.959). The arithmetic mean heights of the two measurements were used for calculation. The anterior-posterior ratio, the middle-posterior ratio, the posterior-posterior ratio were calculated. Prevalent morphometric vertebral deformities (MVD) were defined according to the Genant grading [29]. Vertebral deformities were classified into three types (wedge, biconcavity, crush) and three grades (mild (any ratio <20%), moderate (any ratio between 25–40%), and severe (any ratio >40%)).

The WHO Fracture risk assessment tool (FRAX) was used to calculate the absolute 10-year risk for major and for hip fractures in women and men [12].

Statistical analyses were performed using SPSS version 12.01. Categorical variables and proportions were analyzed using chi-square statistic. Odds ratio (OR) with 95% confidence intervals (95% CI) were calculated based on the chi squares. One way Anova and chi-square statistics were used to analyze differences in continuous variables between subgroups. Observations were considered significant if two-sided p-values were < 0.05.

## Results

Of the 100 patients, 73 were women and were 27 men. Mean age was 68 years (standard deviation: 10 years, range: 50 to 90 years). Demographic data are summarized in Table 1. The majority of patients were Caucasian (97%). Fractures were found at the following locations: clinical vertebral fractures (n = 4), clavicle (n = 3), pelvis (n = 2), humerus (n = 10), radius and/or ulna (n = 24), hand (n = 6), hip (n = 17), tibia/fibula/patella (n = 8) and foot (n = 21). Five patients had multiple simultaneous fractures and 80 patients had fractures after a fall from standing height.

**Table 1:** Characteristics of the patient population (N = 100)

**Variable Median+/-SD**	**All patients**	**Women**	**Men**	**Fragility fracture**	**High energy trauma**	**With contributors**	**Without contributors**
Number	100	73	27	80	20	57	43
Women/men (n)	73/27	na	na	66/14	7/13**	40/17	33/10***
Caucasian ethnicity (n)	97	94	27	79	18	55	42
							
Age (years)	68 ± 10	70 ± 9	62 ± 8*	69,1 ± 9	63.3 ± 9**	71 ± 10	64 ± 7***
Weight (kg)	66 ± 13	63 ± 13	73 ± 11*	65,4 ± 14	68.8 ± 11	64 ± 14	69 ± 11
Spine T-score	-2.88 ± .91	-2.94 ± 0.79	-2.73 ± 1.17	-2.9 ± 0.92	-2.8 ± 0.83	-2.85 ± 0.97	-2.93 ± 0.82
Hip T-score	-1.92 ± 0.9	-2.13 ± 0.92	-1.37 ± 0.70*	-2 ± 0.87	-1.6 ± 1.09	-2.1 ± 1.00	-0.66 ± 0.63***
Hip Z-score	-1.92 ± 0.8	-0.57 ± 0.85	-0.89 ± 0.73	-0.62 ± 0.85	-0.83 ± 0.72	-0.66 ± 0.97	-0.66 ± 0.63
BMD spine(g/sq cm)	0.772+/- 0.100	0.756 ± 0.085	0.810 ± 0.130*	0.765 ± 0.103	0.795 ± 0.094	0.776 ± 0.109	0.766 ± 0.091
BMD hip(g/sq cm)	0.718+/- 0.395	0.676 ± 0.115	0.825 ± 0.107*	0.700 ± 0.116	0.779 ± 0.160**	0.695 ± 0.143	0.749 ± 0.106
Calcium intake(mg/day)	852 ± 432	828 ± 448	915 ± 389	851 ± 467	854 ± 266	744 ± 343	993 ± 497***
Serum 25OH vitamin D (nmol/L)	66 ± 53	67 ± 60	63 ± 28	63 ± 57	75 ± 30	42	0
Creatinine clearance (ml/min)	67 ± 23	62 ± 20	65 ± 23	65 ± 23	74 ± 26	45 ± 18	82 ± 24***
Fracture after fall from standing height (n)	80	66	14	na	na	45	35
N of contributors (n)	86	62	24	70	16	na	na
N with contributors (n)	57	40	17	45	12	na	na
N with bone-related fracture risks	54	42	12	44	10	33	21
N with fall-related fracture risks	79	57	22	65	14	46	33
Time Go Up and Go (min)	8.6 ± 7.9	8.4 ± 8.0	8.9 ± 8.0	8.2 ± 8.0	9.8+/-8.0	8.1+/-8.6	9.2+/-7.1
N with MVD <0.80 (n/n measured)	53/93	35/66	18/27	42/73	11/20	36/54	17/39***
N with MVD <0.75	29	22	7	24	11	19	10

*p < 0.05 women vs. men, ** p < 0.05 between fragility fracture and high-energy trauma, *** p < 0.05 between group with and without contributor, Na: not applicable

A total of 86 contributors to secondary osteoporosis were diagnosed in 57 patients (Table 1 and 2). Contributors consisted of known medical conditions (34 in 27 patients) or newly diagnosed (52 in 50 patients). Seven patients had only known contributors, 20 had known plus a newly diagnosed contributor and 30 had only newly diagnosed contributors. One contributor was found in 32 (of whom 24 were vitamin D deficient), more than one contributor in 25 and 43 had none.

**Table 2:** Contributors to secondary osteoporosis identified in men and women >50 years with a recent clinical fracture (N = 100)

**Contributors**	**Total**	**Known**	**Newly diagnosed**	**Fragility Fracture ** **(N = 80)**	**High-energy trauma ** **(N = 20)**
Endocrine disorders					
Serum 25-OHD_3 _s ≤50 nmol/l	42	0	42	37	5
Hyperparathyroidism secondary to low calcium intake	2	0	2	1	1
Hyperthyroidism	3	3	0	3	0
Hypogonadism (in men)	1	1	0	0	1
Anorexia nervosa (in women)	2	2	0	2	0
Diabetes mellitus	5	5	0	4	1
Gastrointestinal disorders					
Lactose intolerance	1	0	1	0	1
Connective tissue disorders					
Rheumatoid arthritis	2	2	0	1	1
Giant-cell arteritis	1	1	0	1	0
Renal disorders					
Renal insufficiency without secondary hyperparathyroidism	11	5	6	7	4
Renal insufficiency with secondary hyperparathyroidism	3	3	0	3	0
Miscellaneous					
Severe immobility	3	3	0	3	0
Pulmonary diseases	5	5	0	4	1
Medication and life style					
Exogenous hyperthyroidism	1	0	1	1	0
Alcohol abuse	4	4	0	2	2
**Total number of contributors (N = 86)**	**86**	**34**	**52**	**69**	**17**
**Total number of patients with contributors to osteoporosis (N = 57)**	**57**	**27**	**50**	**45**	**12**

**Figure 2:**
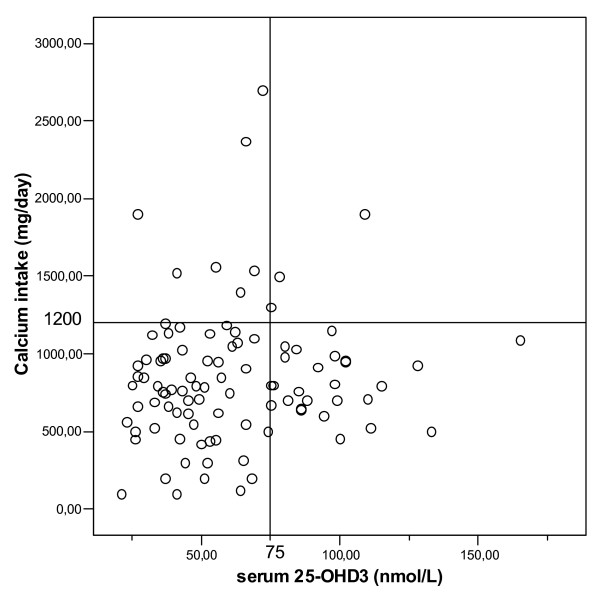
**Calcium intake and serum serum levels of 25OHD3**. Only 3 patients had sufficient intake of calcium and normal serum levels of 25-OHD3.

Based on serum levels of 25-OHD_3_, 11 patients had severe deficiency, 31 were deficient and 31 had insufficient serum values. All were newly diagnosed. Serum levels of 25-OHD_3 _could not be predicted by any of questions on vitamin D or by the sum of those questions. Calcium intake below 1200 mg was reported in 86 patients. Only three patients had both a calcium intake above 1200 mg and a serum 25-OHD_3 _level above 75 nmol/L (Figure 2).

Five patients had secondary hyperparathyroidism, of which four were newly diagnosed (Table 2). Hyperparathyroidism was secondary to renal insufficiency in three cases and to low calcium intake in two cases. We found 14 patients with renal insufficiency, of which 6 were newly diagnosed. Three patients were known with hyperthyroidism, 1 new case of exogenous hyperthyroidism and 1 new case of hypothyroidism was detected. One new case of lactose intolerance was diagnosed. Further contributors included anorexia nervosa in 2 women, documented hypogonadism in one men, pulmonary diseases in 5 patients (chronic obstructive lung disease and asthma), alcohol abuse in 4 men, inflammatory rheumatic diseases in 3 patients (2 with rheumatoid arthritis and 1 with giant cell arteritis) and 3 with severe immobility. Most of these patients did not receive preventive measures for osteoporosis prior to the fracture and were thus not recognized as having a contributor to secondary osteoporosis before the fracture occurred.

Other laboratory abnormalities that required further exploration were found in 14 patients (18 abnormalities in total), including exogenous hypervitaminosis D (n = 1), hypercalciuria (n = 3), TSH outside normal ranges (n = 13) and low serum testosterone in one men (Table 3). Among the 9 patients being treated for hypothyroidism, one was over-treated while three were under-treated based on abnormal serum TSH levels. Among the 3 patients being treated for hyperthyroidism, two were under-treated while one was over-treated.

**Table 3:** Laboratory abnormalities that required further exploration in men and women more than 50 years of age with a recent clinical fracture (N = 100)

Laboratory abnormality	Total
Exogenous hypervitaminosis D (>220 nmol/l)	1
Hypercalciuria in 24 hours urine	3
TSH 0.4–3.5 mU/L	13
- >3.5 mU/L	10
- treated hypothyroidism	9
-TSH <0.4 mU/L	1
-TSH >3.5 mU/L	3
- treated hyperthyroidism	3
-TSH >3.5 mU/L	1
-TSH <0.4 mU/L	2
Serum testosterone in men <12 nmol/L (one measurement)	1

**Total number of patients**	14

According to the Dutch guideline for osteoporosis 54 patients had clinical bone-related risk factors for fractures in addition to their current fracture (Table 4). A history of an additional clinical fracture after the age of 50 was present in 31 patients (two with a previous clinical spine fracture). Additionally, 12 had a mother that had suffered one or more fractures, 3 were severely immobilised, and 23 had a low body weight (60 ≤kg). One bone-related risk factor was found in 41 patients, 2 in 12 and 3 in one patient. According to the Dutch guideline for fall prevention, we found fall related risk factors in 79 of the patients: 22 patients had one risk factor, 21 had two risk factors, and 36 had more than two risk factors. An overlap between clinical bone related risk factors and fall related risk factors was present in 45 patients. The prevalence of clinical bone-related and fall-related risk factors was similar between patients that had documented contributors secondary osteoporosis and those who did not (50% versus 59% for clinical bone related risk factors and 79% versus 84% for fall-related risk factors for fractures).

**Table 4:** Clinical risks for fractures recorded in patients with a recent clinical fracture according to the Dutch guidelines

**CLINICAL RISKS OF FRACTURE**	**Total**	**Women**	**Men**	**Fragility fracture**	**High trauma**	**Contributors**	**No contributors**
Numbers of patients	100	73	27	80	20	57	43
BONE RELATED RISK FACTORS	54	42	12	44	10	33	21
History of clinical fracture after 50 years	31	23	8	25	6	19	12
History of clinical vertebral fracture	2	1	0	1	0	1	0
Mother with fracture	12	9	3	10	2	8	4
Low body weight (<60 kg)	23	20	3	18	5	15	8
Severe immobility	3	3	0	3	0	3	0
Glucocorticosteroids user	0	0	0	0	0	0	0
FALL RELATED RISK FACTORS	79	57	22	65	14	46	33
Mobility: Time Get up and Go test	24	21	3	20	4	12	12
Previous falls: 2 or more falls in the previous year	27	22	5	23	4	15	12
Medication use (benzodiazepines, antiepileptics)	16	14	2	14	2	13	3
Low activities of daily living	49	38	11	43	16*	34**	15
Osteoarthritis	48	40	8	44	4*	25	23
Snellen score-visual acuity less than 0.4	16	8	8	14	2	13	3
Urinary incontinence	19	17	2	19	0	13	6

*p < 0.05 between fragility fracture and high-energy traum fracture, **p < 0.05 between groups with and without contributors

VFA could be performed for 93 patients. Lateral spine images were not available in 7 cases due to severe scoliosis or other technical difficulties such as positioning patients with humerus fracture on the DXA table. On VFA, 57% of patients had a MVD, 31% had more than one MVD and 31% had moderate and severe MVD.

**Figure 3:**
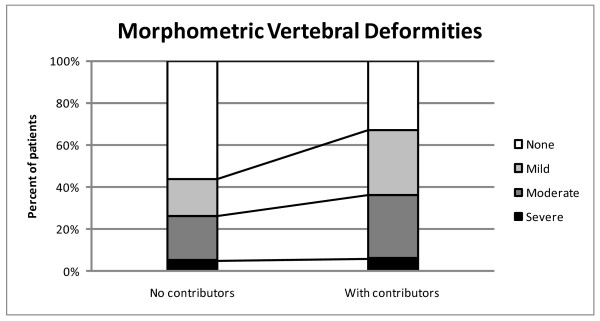
Prevalence of MVD defined according to the grading of Genant et al. in patients with contributors to secondary osteoporosis and in patients without contributors.

The 57 patients with contributors to secondary osteoporosis were older (71 versus 64 yrs, p < 0.01) (Table 1). They had more of some fall risks (multi-medication use (13 versus 3, p < 0.05), restricted activities of daily living (34 versus 15, p < 0.05) and disturbed vision (13 versus 3, p < 0.05) (Table 4)). They had lower calcium intake (744 versus 993 mg, p < 0.05) (Table 1) and more MVD (67% versus 44%, p < 0.05, OR: = 2.6, 95% CI: = 1.1–6.0) (Figure 3).

In contrast, the proportions of women (70 versus 77%) and of patients with fragility fractures (79 versus 81%) were similar between patients with and without contributors. There were also no differences in the prevalence of bone-related clinical risks (59 versus 50%).

Based on the FRAX tool, patients with contributors had a higher calculated absolute 10-year risk for major (16.5 vs. 9.9%, p < 0.01) and for hip fractures (6.9 vs. 2.4%, p < 0.01).

Compared to patients with a high-energy trauma, patients with fragility fractures were older (69 versus 63 years), had better activities of daily living (43 versus 16 patients), and more osteoarthritis (44 versus 4 patients) (Table 1 and 3).

## Discussion

In the 100 patients older than 50 years presenting with a recent clinical fracture and osteoporosis and referred by the surgeons to the rheumatologists in collaboration with the endocrinologists the prevalence of contributors to secondary osteoporosis was high: almost two out of three patients had one or more contributors most of which were correctable.

Our results show that many patients (27%) had known contributors to secondary osteoporosis, a percentage similar to that of Tannenbaum et al. in women with postmenopausal osteoporosis who were seen in an osteoporosis referral centre (32%) [3]. The categories of known contributors to secondary osteoporosis were globally similar as reported by Tannennbaum [3] (endocrine, gastrointestinal and inflammatory rheumatic and pulmonary diseases, severe immobility, alcohol abuse). One exception was glucocorticoid users who are presumably frequently referred to an osteoporosis clinic, but were not represented in our group of patients. In contrast to Tannenbaum, we performed the laboratory test set also in patients with already known contributors and 20 additional contributors were diagnosed in 20 patients (mainly low 25-OHD3, n = 14). Presumably none of the patients with known contributors had received attention in the context of osteoporosis, as none had osteoporosis treatment or calcium and vitamin D supplements.

In the other, presumably healthy patients without known contributors, laboratory testing identified newly diagnosed contributors to secondary osteoporosis in 30 more patients, mostly vitamin D deficiency and renal disorders. The number of patients with newly diagnosed contributors (50%) was higher than reported by Tannenbaum et al. (33%) and concerned mainly vitamin D deficiency, secondary hyperparathyroidism (to renal insufficiency and to low calcium intake), malabsorption and exogenous hyperthyroidism [3]. In contrast to Tannenbaum, we performed TSH not only in patients with a history of thyroid diseases, but in all patients, and were able to diagnose one new case of hypothyroidism.

Vitamin D status could not be identified by history, despite including four specific questions regarding vitamin D intake. It has been shown that there is only a modest relation between reported vitamin D intake from an extensive dietary questionnaire and serum levels of 25-OHD3 [30]. In our study a wide spectrum of levels of serum 25-OHD3 were found, from severely deficient to normal. There is still no consensus about how much vitamin D supplements are required to normalise serum levels. Some propose a unique dose of 800 IU/day together with 1000–1200 mg calcium/day to achieve 50 nmol/L [31,32]. Others state that a unique dose of 800–1600 IU/day would normalize serum levels to >75 nmol/L [33,21]. As patients with low serum levels of 25-OHD_3 _require, at least temporarily, high doses of vitamin D supplements while those with normal levels require less or none [34], measuring serum 25-OHD3 levels is helpful in patients with osteoporosis in order to decide about appropriate vitamin D supplementation [3].

The calcium homeostasis was further compromised by the low calcium intake (<1200 mg/day) in most patients, resulting in secondary hyperparathyroidism in 2, and only 3% of the patients had both adequate calcium intake and vitamin D status. Correcting these combined deficiencies has been demonstrated to reduce fracture risk, at least in institutionalized elderly women [35] and to reduce the risk of falls [36]. Calcium and vitamin D supplementation are thus needed in most patients presenting with a fracture and osteoporosis. However, supplementation with calcium and vitamin D alone is an insufficient measure in patients with osteoporosis, as drug therapy for osteoporosis has been shown to reduce the risk of fractures on top of correcting such deficiencies. Our data, together with those of Edwards et al. [6] indicate that calcium and vitamin D deficiency is frequently present in patients presenting with a fracture, and that these deficiencies need to be identified and corrected.

Interestingly, the presence of contributors was similar between women and men, and between patients with fractures associated with low or high-energy trauma, suggesting that evaluation for secondary contributors is indicated in women and men and after low or high-energetic trauma.

An additional 14 patients had laboratory abnormalities that required further investigation, mainly hypercalciuria, uncontrolled treatment of thyroid disorders and low testosterone (in one man). Hyperthyroidism, whether endogenous or exogenous, can increases bone turnover and contributes to secondary osteoporosis [37,38]. Hypothyroidism on the other hand increases the risk of fractures through low bone turnover if untreated or high bone turnover if over treated [39]. Thus fine-tuning thyroid treatment is indicated. The same is probably true for patients with hypercalciuria in whom thiazides are indicated [40], and for hypogonadism in men that can be treated with testosterone supplementation [41], although no fracture prevention data are available in these conditions.

Therefore, measuring serum 25-OHD_3_, calcium in 24 hours urine, serum creatinine, TSH, PTH as proposed by Tannenbaum et al. is indicated in patients with osteoporosis and a recent clinical fracture, and enabled us to identify 47 (96%) newly diagnosed contributors and 13 of the 14 laboratory abnormalities [3]. As many patients had endocrine diseases, collaboration with endocrinologists appeared to be highly valuable for diagnosis and treatment.

The prevalence of clinical bone-related fracture risks in postmenopausal women, as evaluated by the Dutch guidelines, was similar between patients with and without documented contributors to and it contributed to further specify the risk for fractures.

Nearly 80% of patients had fall-related risk factors for fractures, as reported by others [16]. Although it has not been shown until now that fall prevention strategies itself can prevent fractures, they reduce the risk of falls. [42] A multidisciplinary, multifactorial intervention program reduces postoperative falls and injuries after femoral neck fracture and are therefore applied in our ongoing prevention program [43].

An interesting finding was the prevalence of MVD which was more than twice as high among patients with documented contributors for secondary osteoporosis compared to those without contributors, in spite of similar low BMD in both groups. MVDs, that are related to future fracture risk independent of BMD [11], reflect bone failure independently of BMD and thus indicate other mechanisms of bone's decreased resistance to fracture than low BMD, such as changes in the bone turnover, alterations in micro architecture of bone and deficient mineralization, especially in the context of the high prevalence of calcium and vitamin D deficiency.

In several recent publications differential diagnosis and search for contributors to secondary osteoporosis is advocated [44,45]. Only limited data are available about collaboration between surgeons and internists in taking care for osteoporosis in patients presenting with a fracture. Some initiatives were very successful [46], but in most instances the collaboration is failing [47]. This study indicates that such collaborations add to better treatment of patients with a clinical fracture.

This study has several limitations. Smoking history, which is part of the WHO FRAX tool, was not recorded as it is not part of the Dutch guideline. The sample size was relatively small, but the strength of the study was that consecutive patients were evaluated showing that even in a small group many contributors to secondary osteoporosis could be diagnosed. Some laboratory abnormalities needed further exploration, but were not followed up and so no definite diagnosis could be reported in these patients. VFA has several limitations. Not all vertebrae could be measured, mainly at the upper thoracic level. Identifying patients with MVD by VFA requires additional X-rays to differentiate deformities due to other conditions, such as Scheuerman's disease, degenerative changes or non-osteoporotic short vertebral height. However, the method has a high negative predictive value in predicting the absence of vertebral fractures on X-rays [27]. Another limitation is that only patients with BMD-osteoporosis were included. Most patients with a fracture have no BMD-osteoporosis. The results of our study suggest that documentation of the prevalence of contributors to secondary osteoporosis should also be studied in patients with a clinical fracture without BMD osteoporosis.

## Conclusion

We conclude that more than one in two patients presenting with a clinical vertebral or non-vertebral fracture and BMD-osteoporosis have secondary contributors to osteoporosis, most of which were correctable. Identifying and correcting these associated disorders will enhance treatment effect aimed at reducing the risk of subsequent fractures in patients older than 50 years.

## Abbreviations

BD: Bianca Dumitrescu; BMD: Bone mineral density; CI: confidence interval; DXA: Dual X-Ray absortiometry; ESR: Erythrocyte sedimentation rate; EULAR: European League against Rheumatism; FRAX: Fracture risk assessment tool; MEC: Medical Ethical Committee; MVD: Morphometric vertebral deformity; MVF: Morphometric vertebral fracture; OR: Odds ratio; PTH: Parathormone; TSH: Thyroid stimulating hormone; VFA: Vertebral fracture assessment; WHO: World Health Initiative.

## Competing interests

The authors declare that they have no competing interests.

## Authors' contributions

BD analyzed clinical and laboratory data for the diagnosis of contributors to secondary osteoporosis, performed vertebral fracture assessment, statistical analyses and wrote the manuscript. SvH implicated in the coordination of the study, involved in the treatment of patients included in the study, participated to sequence alignment and data presentation. RtB involved in the coordination of the study, involved in the treatment of patients included in the study. AN–K analyzed clinical and laboratory data for the diagnosis of contributors to secondary osteoporosis, coordinated data presentation. CW gathered laboratory and clinical data, performed statistical analysis. GU participated in the sequence alignment. SvdL analyzed clinical and laboratory data for the diagnose of contributors to secondary osteoporosis, coordinated data presentation. PG conceived the study, participated in the design of the study, coordinated the study, analyzed the data for correct diagnosis and drafted the manuscript.

## Pre-publication history

The pre-publication history for this paper can be accessed here:


